# World Health Organization (WHO) antibiotic regimen against other regimens for the treatment of leprosy: a systematic review and meta-analysis

**DOI:** 10.1186/s12879-019-4665-0

**Published:** 2020-01-20

**Authors:** Maria Lazo-Porras, Gabriela J. Prutsky, Patricia Barrionuevo, Jose Carlos Tapia, Cesar Ugarte-Gil, Oscar J. Ponce, Ana Acuña-Villaorduña, Juan Pablo Domecq, Celso De la Cruz-Luque, Larry J. Prokop, Germán Málaga

**Affiliations:** 1 CONEVID Unidad de Conocimiento y Evidencia, School of Medicine “Alberto Hurtado”, Universidad Peruana Cayetano Heredia, Av. Honorio Delgado 430, Lima 31, Peru; 2 Division of Tropical and Humanitarian Medicine, Geneva University Hospitals and University of Geneva, Geneva, Switzerland; 3 Department of Pediatrics, Mayo Clinic Health System, Mankato, MN USA; 4 Instituto de Medicina Tropical Alexander von Humboldt, Universidad Peruana Cayetano Heredia, Lima, Peru; 5 Montefiore Medical Center-Albert Einstein College of Medicine, Bronx, New York, USA; 6 Division of Pulmonary and Critical Care Medicine, Mayo Clinic, Rochester, MN USA; 7 Cardiology division, Jackson Memorial Hospital, Miami, Florida, USA; 8 Evidence-based Practice Center, Mayo Clinic, Rochester, MN USA; 9 School of Medicine “Alberto Hurtado”, Universidad Peruana Cayetano Heredia, Lima, Peru; 10 Hospital de la Santa Creu i Sant Pau, Institut d’Investigació Biomèdica Sant Pau, and Universitat Autònoma de Barcelona, Barcelona, Spain; 11 Faculty of Infectious and Tropical Diseases, London School of Hygiene and Tropical Medicine, London, UK

**Keywords:** Leprosy, Treatment, Systematic review, World Health Organization

## Abstract

**Background:**

To evaluate the effectiveness and safety of the World Health Organization antibiotic regimen for the treatment of paucibacillary (PB) and multibacillary (MB) leprosy compared to other available regimens.

**Methods:**

We performed a search from 1982 to July 2018 without language restriction. We included randomized controlled trials, quasi-randomized trials, and comparative observational studies (cohorts and case-control studies) that enrolled patients of any age with PB or MB leprosy that were treated with any of the leprosy antibiotic regimens established by the WHO in 1982 and used any other antimicrobial regimen as a controller. Primary efficacy outcomes included: complete clinical cure, clinical improvement of the lesions, relapse rate, treatment failure. Data were pooled using a random effects model to estimate the treatment effects reported as relative risk (RR) with 95% confidence intervals (CI).

**Results:**

We found 25 eligible studies, 11 evaluated patients with paucibacillary leprosy, while 13 evaluated patients with MB leprosy and 1 evaluated patients of both groups. Diverse regimen treatments and outcomes were studied. Complete cure at 6 months of multidrug therapy (MDT) in comparison to rifampin-ofloxacin-minocycline (ROM) found RR of 1.06 (95% CI 0.88–1.27) in five studies. Whereas six studies compare the same outcome at different follow up periods between 6 months and 5 years, according to the analysis ROM was not better than MDT (RR of 1.01 (95% CI 0.78–1.31)) in PB leprosy.

**Conclusion:**

Not better treatment than the implemented by the WHO was found. Diverse outcome and treatment regimens were studied, more statements to standardized the measurements of outcomes are needed.

## Background

Leprosy is a neglected disease caused by *Mycobacterium leprae* that affects the skin and peripheral nerves. Although treatment exists, access is limited and if it is not initiated early in the course of the disease, the control is suboptimal and permanent disabling sequelae can occur [1]. On the other hand, research on leprosy is scarce, limiting the development of new strategies. Additionally, in the general population leprosy is feared and misunderstood. Patients, who suffer this disease, since biblical times, are victims of ostracism, stigma and neglect in many cases [2].

Even when the prevalence of this disease has significantly decreased, and most previously highly endemic countries have reached erradication (defined as a registered prevalence rate of < 1 case/10000 population), it continues to be a global health concern [3]. In 2014, the World Health Organization (WHO) estimated that 213,899 new patients were diagnosed globally. Most of them come from developing countries such as Brazil, India and Indonesia. These nations accounted for 81% of new cases [4].

The best treatment regimen available continues to be controversial with limited evidence support. In 1981, the WHO developed, by consensus, a multidrug therapy (MDT) with dapsone and rifampin for 6 to 12 months for paucibacillary (PB) while clofazimine was added and the length of therapy extended to 24 months for multibacillary (MB) leprosy [5]. These recommendations were reformulated in 1998, reducing the treatment duration to 6 and 12 months respectively, mainly based on economic reasons [6]. On the other hand, the National Hansen’s Diseases Programs (NHDP) in Peru continues to favors a longer duration of therapy [7]. Recently, other drugs as minocycline, ofloxacin, levofloxacin, clarithromycin and moxifloxacin have shown to be effective against *M. leprae* [8].

Considering the lack of an accurate quantitative endpoint (noncultivable pathogen), and a very long observation period for identification of relapse (approximately 15 to 20 years), conducting a trial to compare antibiotic regimens in this disease results extremely difficult, leading to a weakness and lack of evidence. Therefore, we conducted a systematic review to evaluate the effectiveness and safety of the WHO antibiotic regimen for the treatment of PB and MB leprosy compared to other available regimens.

## Methods

This review was reported following the Systematic Reviews and Meta-Analyses (PRISMA) guidelines [9].

### Eligibility criteria

Following our predesign protocol we included randomized controlled trials (RCTs), quasi-randomized trials, and comparative observational studies (cohorts and case-control studies) that enrolled patients of any age with PB or MB leprosy who were treated with any of the leprosy antibiotic regimens established by the WHO in 1982 and used any other antimicrobial regimen as a controller.

### Information sources and search

An experienced librarian (LJP) with expertise in conducting systematic reviews designed and conducted the electronic search strategy with input from the study investigators. We searched multiple electronic databases (Ovid Medline In-Process & Other Non-Indexed Citations, Ovid MEDLINE, Ovid EMBASE, Ovid Cochrane Database of Systematic Reviews, Ovid Cochrane Central Register of Controlled Trials, Scopus, and LILACS) from 1982 to July 2018 without any language restriction. Controlled vocabulary supplemented with keywords was used to search for the topics of leprosy and WHO multidrug therapy (MDT), as well as to limit the search to randomized trials and observational studies conducted in humans. The detailed search strategy is listed in Additional file 1. This search was complemented by a manual search that included, reviewing the reference lists of the eligible primary studies, narrative reviews and queried experts.

### Study selection

Two independent reviewers screened all abstracts and titles and selected potentially eligible studies for full-text assessment. Disagreements were included during this phase. Upon retrieval of the full text version of potentially eligible studies, the review process was repeated using pre-defined eligibility criteria. Disagreements were resolved by consensus (reviewers discussed the study and reached a consensus); when this was not possible, by arbitration (a third reviewer). We achieved almost perfect agreement (k = 0.9) during this phase.

### Data collection

Data were extracted using a pre-designed, piloted extraction form. Working in duplicates and independently reviewers extracted the following variables from each study: study characteristics, baseline patient characteristics, intervention details, and outcomes of interest. Disagreements were resolved by consensus.

Primary efficacy outcomes included: 1) Complete clinical cure, defined as full regression of the lesions; 2) Clinical improvement of the lesions, defined by a clinical criteria; 3) Relapse rate, defined as the presence of the disease after completing the treatment (clinical or bacteriological or therapeutic criteria) [10]; 4) Treatment failure, defined as persistence or worsening of skin lesions and failure to improve the bacillary index for MB leprosy. Bacillary index (BI) improvement and neuritis were considered secondary efficacy outcomes. Neuritis was considered if participants reported pain during the interview or when participants complains of pain in one or more peripheral nerve trunks of the limb(s) during the period of the treatment.

Regarding safety outcome, we evaluated severe side effects (defined as a side effect that forced the patient to stop the treatment), and mild to moderate side effects. Also, we evaluated immunological reactions: type I (reverse reaction), and type II (erythema nodosum leprosorum - ENL).

### Author contact

We contacted corresponding authors of each study twice within 2 weeks via e-mail or by phone or mail when e-mail was not available if additional information was required.

### Quality assessment

We used the Cochrane Collaboration’s tool for assessing risk of bias [11] to evaluate the methodological quality of the included RCTs. This was assessed independently by duplicate for each study. For observational studies, we used the Newcastle-Ottawa Scale [12].

### Statistical analysis and data synthesis

We summarized the qualitative data (eg. study population, design, intervention, comparison, outcomes) of the included studies in tables. If meta-analysis was appropriate, data was pooled using a random effects model to estimate the treatment effects reported as relative risk (RR) with 95% confidence intervals (CI) [13] for dichotomous outcomes and mean difference (MD) or standardized mean difference (SDM) with 95% CI for continuous outcomes. Statistical heterogeneity was assessed using I^2^ statistic [14]; I^2^ values over 50% indicated significant heterogeneity across studies’ populations and interventions. In case data were insufficient for meta-analysis, a descriptive summary of the outcome findings was reported per categorization of type of leprosy (PB and MB). If the primary studies did not report standard deviations so they had to be inputted using the other studies as reference [15].

### Assessment of publication bias

The assessment of publication bias was unfeasible due to the small number of trials [15].

## Results

### Search results and study description

**Fig. 1:**
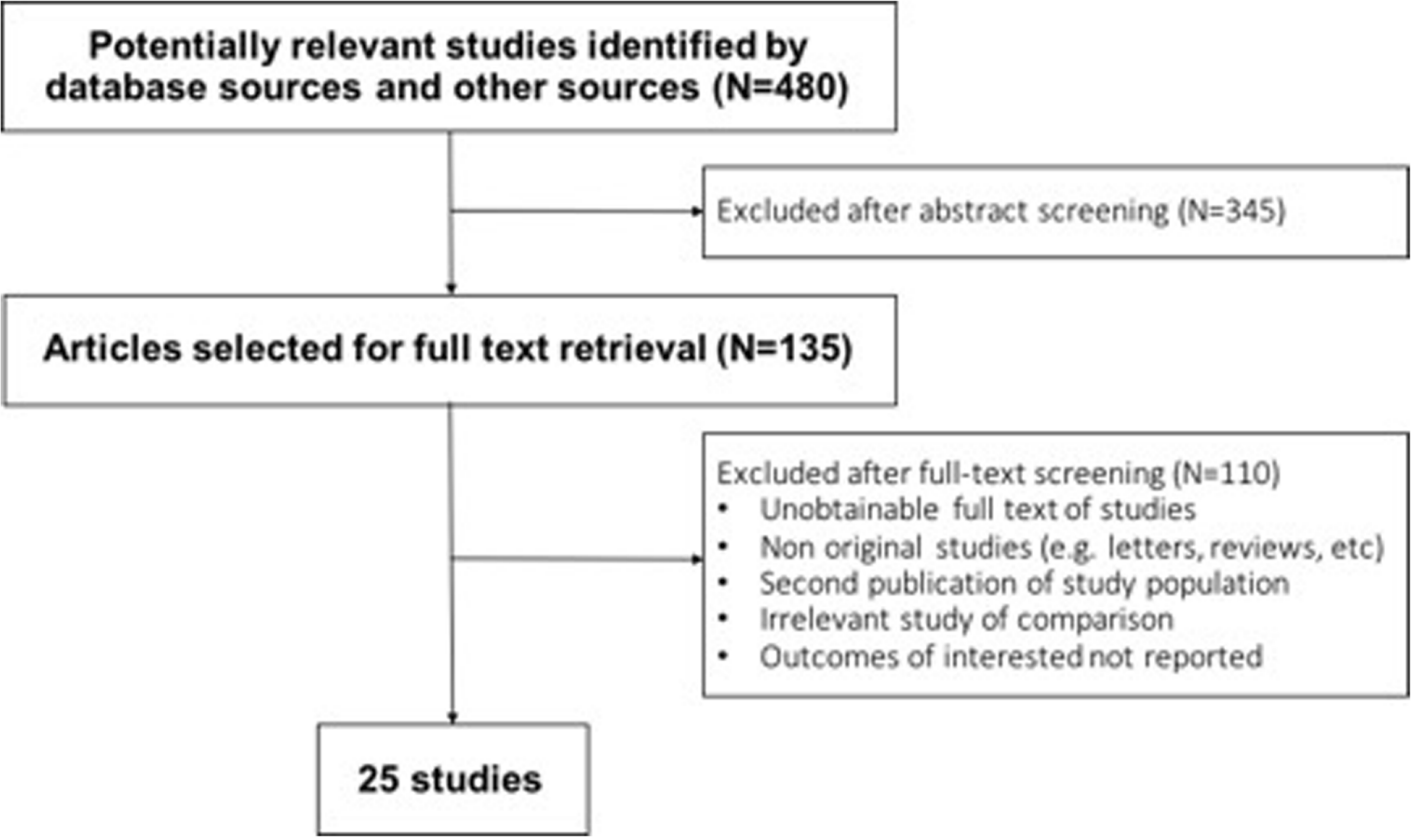
Flowchart of the study

The primary search strategy identified 480 references. After initial screening, 135 studies were eligible to full text screening. Finally, 25 studies (22 RCTs, a case-control study and 2 non-randomized trial) met our inclusion criteria and were included in our review (Fig. 1). One study does not have the full text available and we contacted the corresponding author without getting an answer. Also, another author was contacted to ask the author about the study design without a successful answer. No unpublished studies were identified.

The included studies (Tables 1 and 2) enrolled 8214 patients with a mean age of 35.2 years (range: 6 to 75 years). Eleven studies evaluated patients with PB leprosy (4281 patients), while 9 evaluated patients with MB leprosy (3869) patients) and 1 included patients from both groups. Follow up period ranged from 3 months to 12 years. All the studies were conducted in the developing world, being India the most common country (11 studies).

**Table 1:** Study characteristics in Paucibacillary Leprosy

Study	Study Design	Country	Follow Up (years)	N	Age (Years)	Diagnosis	Control Used	Evaluated Outcome
2–3 LMT, 2001 [16]	RCT	India	1.5	236	Children and adults	Clinical and bacteriological^a^	ROM (single monthly dose, children were given half the dose)	-Clinical score -Lesion resolution -Tx failure
Babu, 1997 [17]	RCT	Multicenter	1.5	1483	Mean 23.5	Clinical and bacteriological^a^	ROM (single monthly dose)	Clinical score
Balagon, 2010 [18]	RCT	Phillipines	12	124	MDT:33.5±13.2 35.1±15.2	NR	28 days of RFP + ofloxacin for 4w then 5 m of placebo	-Relapse -Cure
Bathe,1986 [19]	RCT	India	1	80	19–45	Clinical, bacteriological, immunological, histological	MDT + clofazimine in alternative days for 6 m	Clinical score
Deshmuk, 2003 [20]	RCT	India	0.5	32	23 ≦ 30 years and 9 > 30 years	Clinical and bacteriological^a^	ROM (single monthly dose)	Clinical and histopathological score
Emmanuel, 2005 [21]	RCT	India	2	51	Children and adults	Clinical and histological	ROM (single monthly dose)	Clinical score
Katoch, 1999 [22]	RCT	India	2	300	NR	NR	MDT + clofazimine	-Active disease/signs of activity -Mitsuda reaction -Relapse
Kumar 2015 [23]	RCT	India	8	268	Mean 38.3	Skin lesions and nerve involvement	ROM (Single monthly dose)	Cure and relapse
Manickam 2012 [24]	RCT	India	3	1526	Mean 27	Skin lesions and nerve involvement	ROM (single dose, children were given half the dose)	-Clinical score -Complete clearance
Mathai, 1991 [25]	RCT	India	2	54	7–67	NR	MDT + dapsone	Mitsuda reaction
Orege, 1990 [26]	RCT	Kenya	0.6	127	NR	Clinical	MDT + RFP + dapsone	Clinical score
Rao, 2009 [27]	Not randomized	India	2	32	NR	Clinical	RFP + dapsone + clofazimine × 6 m	Clinical and histopathological score

*Abbreviations*.- *BI* Bacilar index, *MDT* Multidrug Treatment (WHO), *NR* Not reported, *PB* Paucibacilar, *RFP* Rifampin, *ROM* Rifampin-ofloxacin-minocycline, *Tx* Treatment, *RCT* Randomized Controlled Trial

^a^No further details provided

**Table 2:** Study characteristics in Multibacillary Leprosy

Study	Study Design	Country	Follow Up (years)	N	Age (Years)	Diagnosis	Control Used	Evaluated Outcome
Balagon 2011 [28]	Cohort	Philippines	2	589	6–73	NR	MDT 2y	Type II reaction
Bathki, 1992 [29]	RCT	India	2	16	NR	Clinical and smear	MDT + vaccine	BI
Fajardo, 2009 [30]	RCT	Philippines	12	1483	15–64	Clinical and smear	-MDT 1y - 1 month of daily RFP + ofloxacin -MDT 1y + 1-month daily RFP/ofloxacin	-Relapse -Skin smear
Fernandes Pena 2012 [31]	RCT	Brazil	4.9	613	Mean 40.15	NR	Monthly RFP, dapsone and clofazimine daily × 6 m	Type I or II or pure neuritis
Gunawan, 2018 [32]	RCT	Indonesia	0.25	14	41. 71±12.53	BI and smear	clarithromycin+ dapsone+ clofazimine	BI
Jadhav, 1992 [33]	RCT	India	2	88	Mean 24	Smear	RFP daily for 9 months and then 1 month till the end of 2 years + dapsone + clofazimine daily	-BI -Morphological index
Maghanoy, 2018 [34]	RCT	Philippines	2	100	Median 30	NR	MDT + 12 months of clofazimine	Type II reaction
Oliveira Penna. 2017 [35]	RCT	Brazil	5	613	Mean 40.2	NR	RFP + dapsone + clofazimine × 6 m	-BI -Type I and II reactions -Disability -Relapse rate
Rao 2009 [27]	Not randomized	India	2	32	NR	Clinical	RFP + dapsone + clofazimine × 6 m	Clinical and histopathological score
Sampoonachot, 1997 [36]	RCT	Thailand	6	60	12–75	NR	-MDT + ofloxacin. - ofloxacin + clofazimine, then MDT	-BI -Histological improvement
Shaw, 2003 [37]	Not randomized	India	2	44	37.2 ± 14.3	NR	RFP + clofazimine+ acedapsone + dapsone	BI
Souza Cunha 2012 [38]	RCT	Brazil	7 after the end of tx	198	NR	Untreated MB with BI ≧ 2	-MDT 1y + 1 m of ofloxacin -MDT 1y + 1 m of daily ofloxacin -MDT 1y + daily RFP	Relapse
Tejasvi, 2006 [39]	RCT	India	1	30	27.67 ± 13.08 31.50 ± 17.03	NR	RFP+ sparfloxacin + clarithromycin + minocycline	-BI -Morphological index -Histopathology -Clinical evaluation
Villahermosa, 2004 [40]	RCT	Philippines	2. Additional 8y	21	16–59	Clinical and smear (> 1 of 6 smear sites having a BI ≧ 1)	ROM (24 consecutive monthly observed doses of rifampin (600 mg), ofloxacin (400 mg), and minocycline (100 mg).	Lesion resolution

*Abbreviations*.- *BI* Bacilar index, *MB* Multibacilar; *MDT* Multidrug Treatment, *NR* Not reported, *RFP* Rifampin, *ROM* Rifampin-ofloxacin-minocycline *Tx* Treatment; *RCT* Randomized Controlled Trial

Comparison treatments varied between studies. The most common treatment was a single monthly dose of rifampin, ofloxacin and minocycline (ROM) used in 8 studies (6 of PB and 2 of MB leprosy). The outcomes evaluated were also markedly heterogeneous, as it can be seen in Tables 1 and 2.

### Quality assessment

**Fig. 2:**
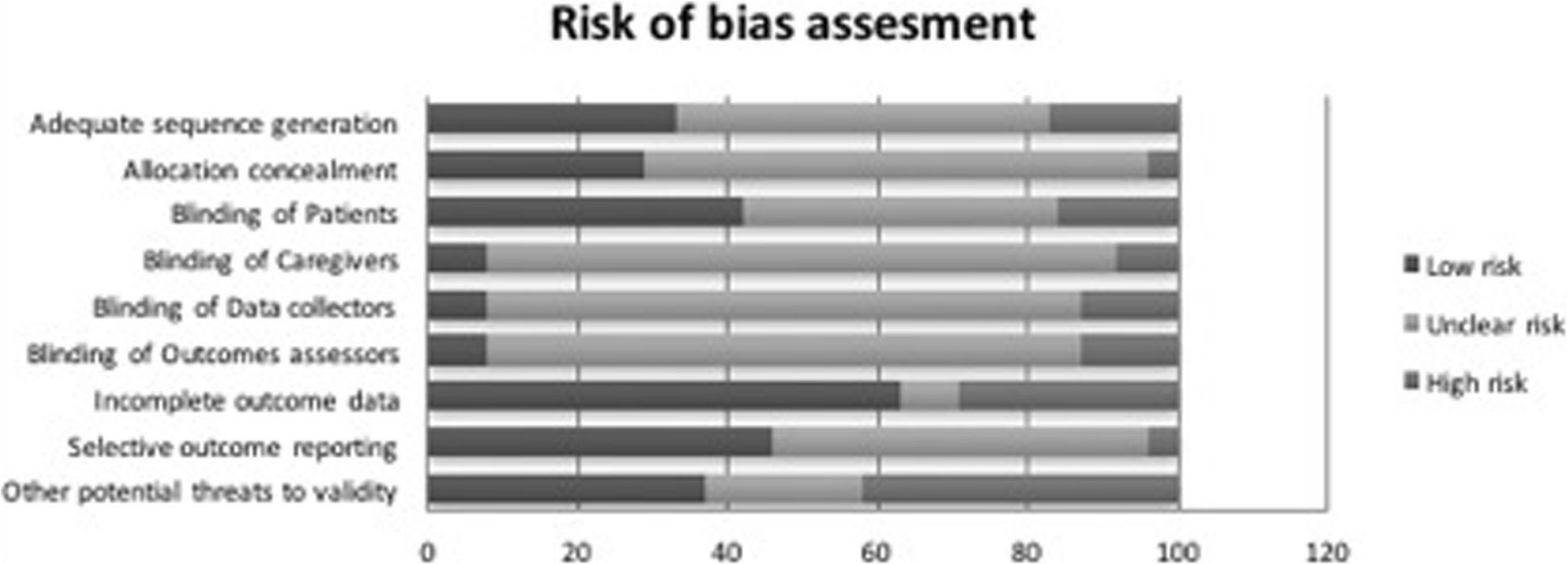
Risk of bias of the selected studies

The overall risk of bias of the included trials was considered moderate to high. The vast majority of the studies did not clearly report the items evaluated. Most of the included trials reported they attrition rate during the follow up (18 out of 20), with a mean of 10.5% (Fig. 2). One observational study was included. Quality was evaluated using the Newcastle- Ottawa scale. Details are presented in Additional file 2.

### Outcomes of interest

#### Efficacy outcomes

##### Complete clinical cure

Complete clinical cure was evaluated in 9 studies that included patients with PB leprosy. It was not evaluated in MB leprosy.

**Fig. 3:**
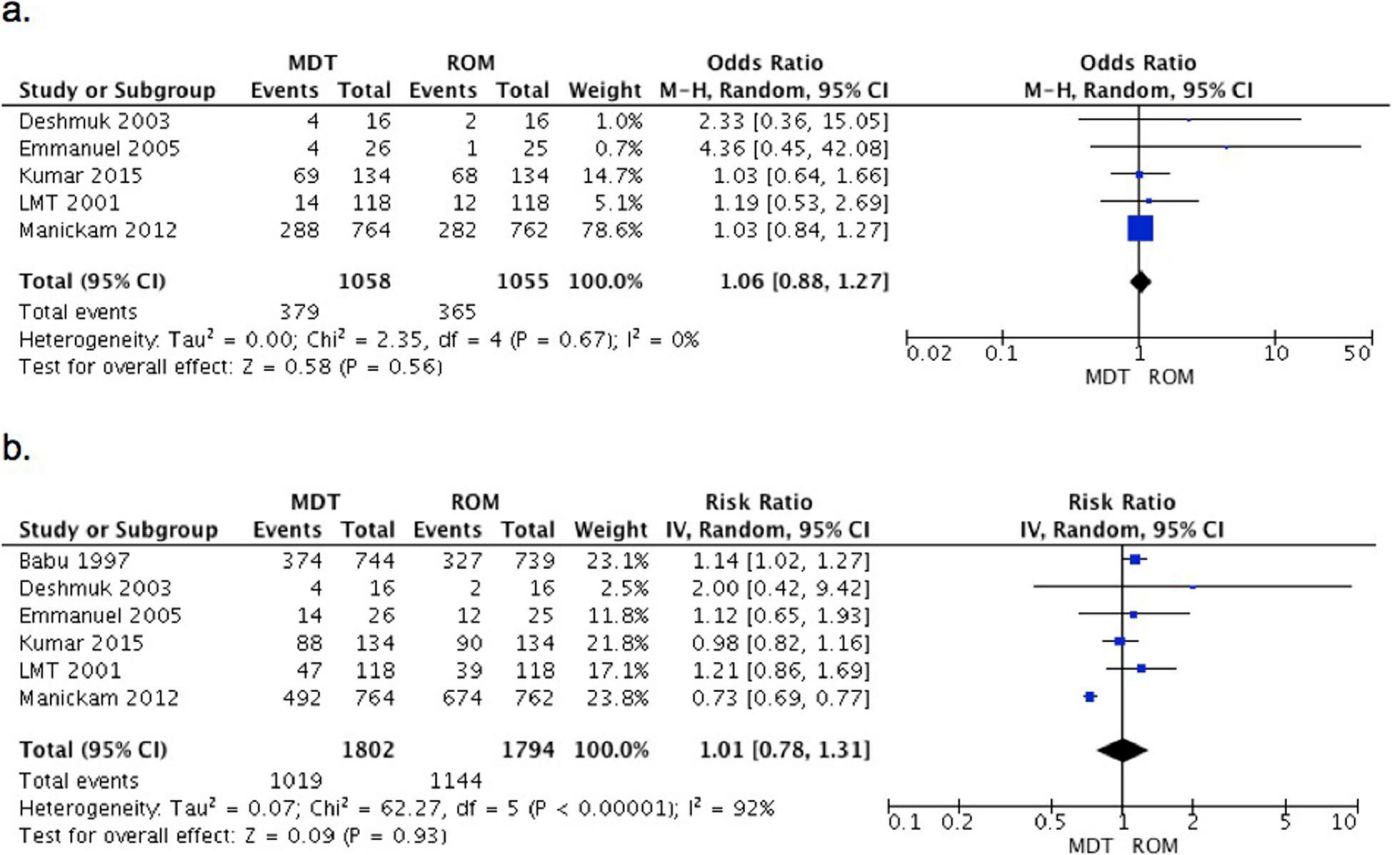
Complete cure in patients with PB leprosy: MDT vs. ROM. **a**. At 6 months **b**. At the end of follow-up period

A meta-analysis of 5 studies comparing MDT vs. ROM did not show statistically significant difference between them after 6 months of treatment (RR 1.06, 95% CI 0.88–1.27, *p* = 0.56). When the same comparison was evaluated at the end of the study period (range 6–36 months) in 6 studies, ROM was not better than MDT (RR 1.01, 95% CI 0.78–1.31, *p* = 0.93). The studies with more participants that were included in the analysis had significant and contrary results with RR of 1.14 (95%CI 1.02–1.27) by Babu et al., 1997 and 0.73 (95% CI 0.69–0.77) by Manickam et al., 2012 (Fig. 3a and b).

The addition of clofazimine to PB MDT did not show significant improvement. This and other available comparisons are shown in Table 3.

**Table 3:** Efficacy Outcomes

Complete Clinical Cure						
Study	Patients	Comparison	Follow up	RR	95% CI	*p* value
Balagon, 2010 [18]	PB	MDT vs. 28 days of RFP + ofloxocin for 4w then 5 months of placebo	6 m	4.2	2.25–7.85	< 0.05
			12 m	0.93	0.80–1.09	0.38
Bathe, 1986 [19]		MDT vs. MDT+ clofazimine in alternative days for 6 m	6 m	1.25	0.77–2.04	0.37
			24 m	1.09	0.93–1.27	0.29
Orege, 1990 [26]		MDT vs. Modified MDT (MDT + RFP + dapsone)^a^	6 m	0.76	0.61–0.95	0.02
Clinical Improvement						
Study	Patients	Comparison	Follow up	MDT	Control	
Rao, 2009 [27]	PB	MDT vs RFP + dapsone + clofazimine (good)	24 m	6/11 (52%)	7/9 (78%)	
		MDT vs RFP + dapsone + clofazimine (moderate)		3/11 (27%)	2/9 (22%)	
	MB	MDT vs RFP + dapsone + clofazimine (good)		13/17 (77%)	1/4 (25%)	
		MDT vs RFP + dapsone + clofazimine (moderate)		3/17 (17%)	None	
Sampoonachut, 1997 [36]		MDT vs. MDT + ofloxacin.	36 m	66.70%	73.30%	
		MDT vs. ofloxacin + clofazimine, then MDT		66.70%	76.50%	
Tejasvi, 2006 [39]		MDT vs. RFP + Sparfloxacin + clarithromycin + minocycline	12 m	66.66%	73.92%	
Villahermosa, 2004 [40]^b^		MDT vs. ROM	96 m	22 points	20 points	
Relapse						
Study	Patients	Comparison	Follow up	RR	95% CI	*p* value
Balagon, 2010 [18]	PB	MDT vs. 28 days of RFP + ofloxacin for 4w then 5 m of placebo	12 m	1.76	0.16–18.88	0.64
Katoch, 1999 [22]		MDT vs. MDT+ clofazimine	24 m	5	0.24–103.28	0.3
Kumar, 2015 [23]		MDT vs. ROM	24 m	0.5	0.05–5.45	0.57
			96 m	1.7	0.94–3.07	0.08
Manickam, 2012 [24]		MDT vs. ROM	6 m	1.43	0.56–3.64	0.46
Fajardo 2009 [30]	MB	MDT 2y vs. MDT 1y + 1 month daily RFP/ofloxacin	144 m	0.51	0.05–5.46	0.58
		MDT 2y vs. 1 month daily RFP/ofloxacin		0.11	0.02–0.87	0.04
		MDT 2y vs. MDT 1 y		0.05	0.01–0.37	< 0.05
Olivera Penna, 2017 [35]		MDT vs. RFP+ dapsone + clofazimine × 6 m	60 m	0.16	0.02–1.29	0.08
Souza Cunha, 2012 [38]		MDT 1y vs. MDT x 2y	84 m	2.59	0.13–52.17	0.53
		MDT 1y vs. MDT + ofloxacin		1.04	0.15–7.10	0.97
		MDT 1y vs. ofloxacin + RFP		0.13	0.03–0.51	< 0.05
Villahermosa, 2004 [40]		MDT vs. ROM	96 m	No events reported		
BI						
Study	Patients	Comparison	Follow up	MD	95% CI	*p* value
Bathki, 1992 [29]	MB	MDT vs. MDT + vaccine	24 m	1.3	0.48, 2.12	< 0.05
Gunawan 2018 [32]		MDT vs. CDC	3 m	0.03	−0.03,0.09	0.75
Jadav, 1992 [33]		MDT vs. RFP + dapsone + clofazimine	24 m	0.41	0.12, 0.70	< 0.05
Olivera Penna, 2017 [35]		MDT vs. RFP+ dapsone + clofazimine × 6 m	60 m	− 0.15	−1.06,0.76	0.34
Sampoonachut, 1997^c^ [36]		MDT vs. MDT + ofloxacin.	36 m	−0.07	− 0.48, 0.34	0.74
		MDT vs. Ofloxacin + clofazimine, then MDT		−0.68	−1.10, − 0.26	< 0.05
Shaw, 2003^c^ [37]		MDT vs. RFP + clofazimine+ acedapsone + dapsone	24 m	0.1	− 0.34, 0.54	0.66

*Abbreviations*. - *BI* Bacillary index, *CDC* Clarithromycin+ dapsone+ clofazimine, *m* Months, *MDT* Multidrug treatment, *ROM* Rifampin, ofloxacin and minocycline

^a^Included a period of direct observation

^b^Maximum Improvement score at the end of treatment; no other information reported

^c^SD not reported

##### Clinical improvement

This outcome was assessed in 5 studies evaluating PB leprosy and in 3 evaluating MB leprosy. All of them used clinical scores that considered the characteristics of the lesions. Evaluated criteria included erythema, hypopigmentation, infiltration, anesthesia, nerve involvement and size. Since the scoring systems were not consistent between studies, we decided to analyze this outcome using standardized mean difference (SMD).

**Fig. 4:**
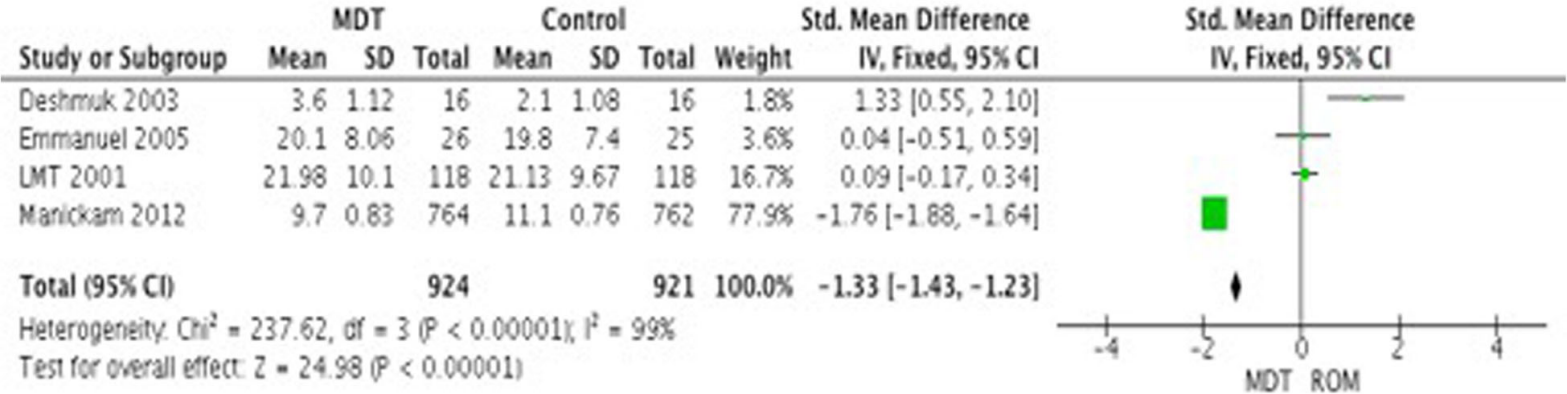
Clinical improvement in patients with PB leprosy: MDT vs. ROM at the end of follow up period

A meta-analysis of 4 studies comparing MDT vs. ROM in patients with PB leprosy showed a difference between both treatments (Standard mean difference − 1.33 (95% CI -1.43- -1.23)). However, this difference was led by one study with significant results and high number of participants (Manickam 2012), additionally the I^2^ show great heterogeneity (Fig. 4). Also, the addition of clofazimine to MDT did not show any significant improvement in this population (SMD 0.28, 95% CI -0.16-0.72, *p* = 0.22).

Two of the studies (Sampoonachut 1997 and Tejasvi 2006) evaluating patients with MB leprosy reported clinical improvement as a dichotomous outcome; none of them reported enough information for the development of a pooled estimated. Villahermosa 2004 reported an improvement score (Table 3).

##### Treatment failure

**Fig. 5:**
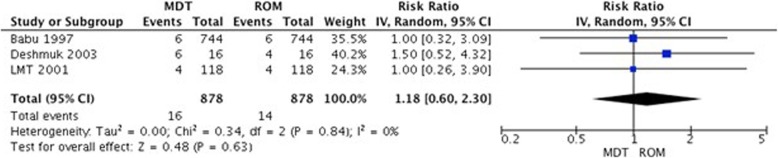
Treatment failure in patients with PB leprosy: MDT vs. ROM at the end of follow up period

Three studies evaluating PB leprosy patients included treatment failure as their outcomes. These studies evaluated MDT vs. ROM in PB leprosy and no statistically significant difference between the treatment arms was found (Fig. 5).

##### Relapse

**Fig. 6:**
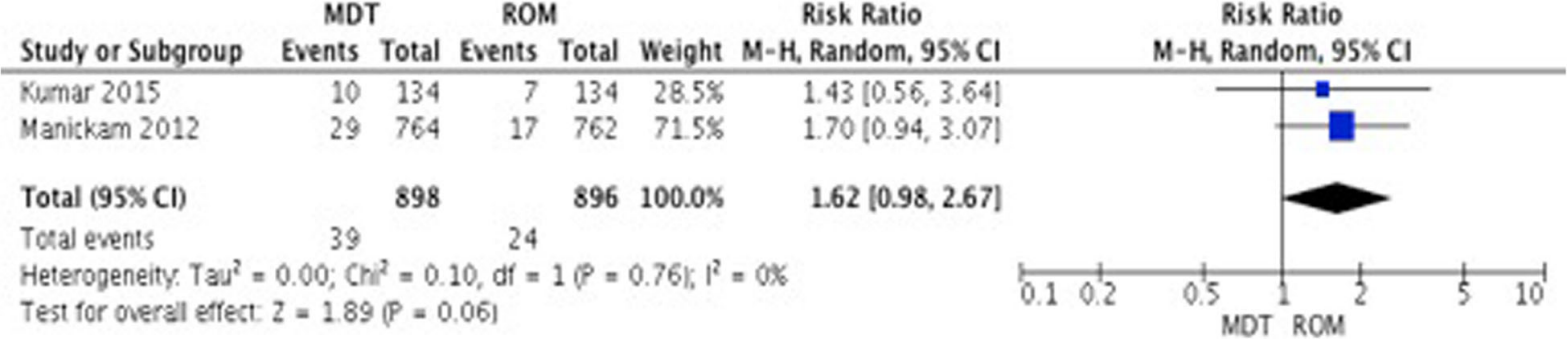
Relapse rate in patients with PB leprosy: MDT vs. ROM at the end of follow up period

This outcome was evaluated in 8 studies. Two studies used ROM as their comparison for patients with PB leprosy and one for MB leprosy. The pooled estimate of the studies in patients with PB leprosy showed no difference between both regimes (RR 1.62, 95% CI 0.98–2.67, *p* = 0.06) but there seem to be a tendency to favor ROM (Fig. 6). The big difference in follow up periods complicates the interpretation of this estimate (6 months vs. 8 years). Additionally, the study evaluating MB leprosy did not report any relapses.

None of the regimes evaluated showed any benefit over MDT for patients with PB leprosy or MB leprosy (Table 3).

##### Bacillary index (BI) improvement

BI was exclusively reported for MB leprosy. Six studies evaluated this outcome. In this case, most of the alternate regimes achieved better reductions of BI compared to MDT. Detailed results are reported in Table 3.

#### Safety outcomes

##### Side effects

We intended to evaluate mild-moderate and severe side effects independently; unfortunately, most studies did not report their outcomes in this way. The development of side effects was analyzed as a single outcome.

**Fig. 7:**
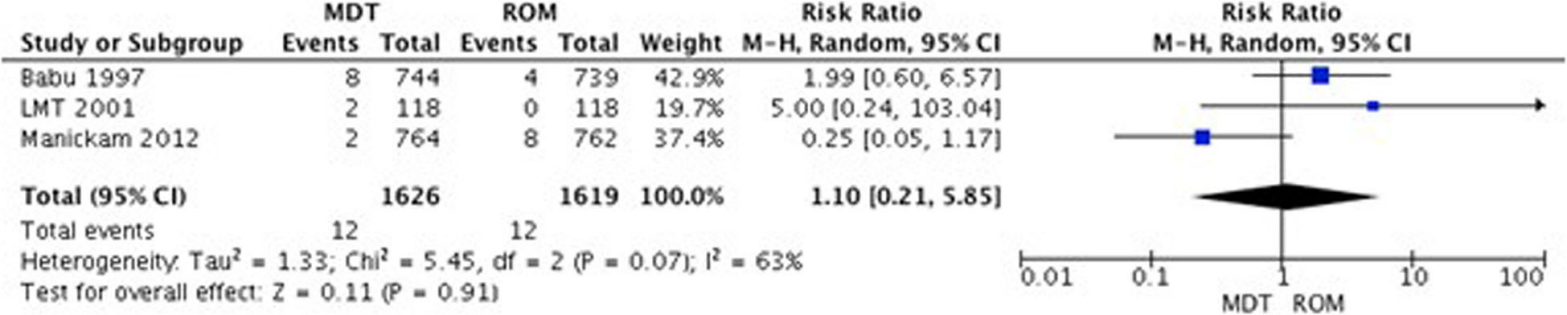
Adverse events in patients with PB leprosy: MDT vs. ROM at the end of the follow up period

Three studies reported side effects in patients with PB leprosy. No statistically significant difference was seen in patients receiving MDT or ROM (Fig. 7).

This outcome was reported in 4 studies evaluating patient with MB leprosy. Each study evaluated a different comparison. Individual estimates are reported in Table 4.

**Table 4:** Safety Outcomes and Immunological Reactions

Side Effects						
Study	Patients	Comparison	Follow up	RR	95% CI	*p* value
Balagon, 2010 [18]	PB	MDT vs. 28 days of RFP + ofloxacin for 4w then 5 m of placebo	12 m	0.38	0.10–1.39	0.14
Gunawan 2018 [32]	MB	MDT vs. CDC	3 m	Unable to analyze		
Tejasvi, 2006 [39]		MDT vs. RFP + sparfloxacin + clarithromycin + minocycline	12 m	0.1	0.01–1.56	0.1
Shaw, 2003 [37]		MDT vs. RFP + clofazimine+ acedapsone + dapsone	24 m	1.42	0.58–3.47	0.44
Type I reaction						
Study	Patients	Comparison	Follow up	RR	95%CI	p value
Orege, 1990 [26]	PB	MDT vs. MDT + RFP + dapsone^a^	6 m	1.15	0.58–2.27	0.69
Fernandes Pena 2012 [31]	MB	MDT vs. Monthly RFP, dapsone and clofazimine daily × 6 m	60 m	1.1	0.86–1.41	0.44
Sampoonachut, 1997 [36]		MDT vs. MDT + ofloxacin	12 m	2.11	0.21–21.36	0.53
		MDT vs. Ofloxacin + clofazimine, then MDT		1	0.16–6.38	1
Shaw, 2003 [37]		MDT vs. RFP + Clofazimine+ Acedapsone + dapsone	24 m	1.78	0.53–5.97	0.35
Tejasvi, 2006 [39]		MDT vs. RFP + sparfloxacin + clarithromycin + minocycline	12 m	0.21	0.01–3.71	0.29
Villahermosa, 2004 [40]		MDT vs. ROM	96 m	0.91	0.37–2.23	0.83
Type II reaction						
Study	Patients	Comparison	Follow up	RR	95% CI	*p* value
Babu, 1997 [17]	PB	MDT vs. ROM	18 m	No events reported		
Balagon, 2011 [28]	MB	MDT 1y vs. MDT 2y	24 m	1.53	1.05–2.23	0.03
Fernandes Pena 2012 [31]		MDT vs. Monthly RFP, dapsone and clofazimine daily × 6 m	60 m	1.15	0.72–1.84	0.56
Jadhav, 1992 [33]		MDT vs. RFP + dapsone + clofazimine	24 m	0.22	0.07–0.72	0.01
Maghanoy, 2018 [34]		MDT vs. MDT + 12 months of clofazimine	24 m	0.86	0.52–1.40	0.54
Sampoonachut, 1997 [36]		MDT vs. MDT + ofloxacin.	24 m	0.7	0.13–3.75	0.68
		MDT vs. ofloxacin + clofazimine, then MDT		1	0.16–6.38	1
Shaw, 2003 [37]		MDT vs. RFP + clofazimine+ acedapsone + dapsone	24 m	0.44	0.04–4.45	0.49
Tejasvi, 2006 [39]		MDT vs. RFP + sparfloxacin + clarithromycin + minocycline	12 m	No events reported		
Villahermosa, 2004 [40]		MDT vs. ROM	96 m	0.91	0.16–5.30	0.92
Neuritis						
Study	Patients	Comparison	Follow up	RR	95% CI	*p* value
Manickam, 2012 [24]	PB	MDT vs. ROM	6 m	0.5	[0.05, 5.49]	0.57
Bathe, 1986 [19]		MDT vs. MDT+ clofazimine	24 m	4	[0.47, 34.24]	0.21
Katoch, 1999 [22]		MDT vs. MDT+ clofazimine	24 m	No events reported		
Fernandes Pena 2012 [31]	MB	MDT vs. RFP, dapsone and clofazimine × 6 m	60 m	0.64	0.45, 0.92	0.01
Jadhav, 1992 [33]		MDT vs. RFP + dapsone + clofazimine	24 m	0.29	0.03–2.69	0.28
Sampoonachut, 1997 [36]		MDT vs. MDT + ofloxacin	24 m	2.11	0.21, 21.36	0.53
		MDT vs. ofloxacin + clofazimine, then MDT		1	0.16–6.38	1
Shaw, 2003 [37]		MDT vs. RFP + clofazimine+ acedapsone + dapsone	24 m	1.19	0.31–4.51	0.8

*Abbreviations* - *BI* Bacillary index, *CDC* Clarithromycin+dapsone+clofazimine, *RFP* Rifampin; m: months, *MDT* Multidrug treatment, *ROM*, rifampin, ofloxacin and minocycline

^a^Included a period of direct observation,

#### Immunological reactions

##### Type I reaction

**Fig. 8:**
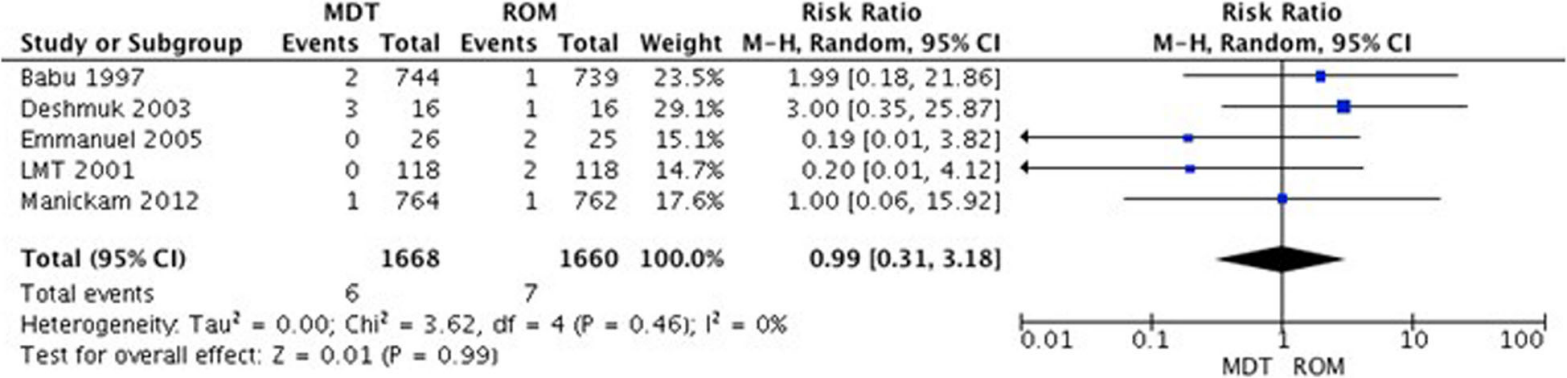
Type I reaction in patients with PB leprosy: MDT vs. ROM at the end of the follow up period

This outcome was evaluated in 11 studies (6 PB and 5 MB). A meta-analysis comparing MDT vs. ROM in patients with PB leprosy did not show a statistically significant difference between the interventions (RR 0.99, 95%CI 0.31–3.18, *p* = 0.99, Fig. 8). Other studies evaluating this outcome are reported in Table 4.

##### Type II reaction

This outcome was evaluated in 9 studies (1 PB and 8 MB). We were not able to develop a meta-analysis. In patients with MB leprosy the combination of dapsone, rifampin and clofazimine showed a statistically significant reduction in the development of type II reaction. On the other hand, the use of MDT regimen for 2 years increased the development of this outcome in the same population when compared to the use of MDT for 1 year. No other statistically significant differences were observed (Table 4).

##### Neuritis

The development of neuritis was evaluated in 7 studies (3 PB and 4 MB). When MDT was compared to rifampin, dapsone and clofazimine for 6 months in patients with MB leprosy a statistically significant difference between groups was seen (Table 4).

## Discussion

### Main findings

This study aimed to summarize all the evidence available on WHO MDT treatment effectiveness, safety and immunological reactions for leprosy. One of the aspects that surprised us the most was to see the poor scientific production for a thousand-year-old disease, the lack of standardization in definition of diagnostic criteria, outcome objectives and time for follow up and if we add to this the poor methodological quality of the majority of studies found, show us a situation that support the denomination and let us understand what leprosy is a neglected disease.

It was precisely this potentially high risk of bias studies that did not let bring a consensus: MDT seems to have a better treatment outcome (clinical improvement) compared with ROM at the end of the follow up period, however we did not find differences in complete cure at 6 months of treatment or at the end of follow up period. When MDT is compared with other treatment, we did not find differences in treatment outcomes.

MDT, as is noted above, had almost no difference in a better response for cured and reduced relapse rate, compared with ROM and other treatments schemes, since most of the magnitudes of these differences are small. However, these schemes had a great success rate after 1 or 2 years of follow-up. These results can confirm the useful role of MDT in leprosy treatment, however also reveals gaps in the evidence to improve it: there is lack of information about adherence in these long-time treatments (MDT, ROM and others), together with the lack of resistance pattern of relapse cases, this successful treatment scheme can be in jeopardized by the increasing rate of relapse cases and the unknown effect of resistance epidemiology of *M. leprae* [8, 41, 42]. Recently the new guidelines of WHO states that “The same 3-drug regimen of rifampicin, dapsone and clofazimine may be used for all leprosy patients, with a duration of treatment of 6 months for PB leprosy and of 12 months for MB leprosy. (Strength: conditional, quality of evidence: low)”. However, they mentioned that “evidence on the potential benefits and harms of a shorter (6-month) 3-drug regimen was limited and inconclusive, with a potential increase in the risk of relapse. Therefore, the Guideline Development Group determined that there was not enough evidence of equivalent outcomes to support a recommendation to shorten the treatment duration for MB leprosy” [43]”. The decision was taken based in only one study [27] which, although it shows an interesting design, its non-randomized and has a very low quality of evidence [44]. Also, the study has not been valued in comparison with other studies, and besides these, its findings had not been supported by a RCT [35].

Is in this context, where supported treatment are required (leprosy programs were reduced their support in the last years) [8], new treatment schemes are needed, considering WHO MDT had more than 30 years of use. However, new treatment should be based in evidence and not only in logistical aspects.

### Limitations and strengths

This study has limitations: primary studies showed high risk of bias, specifically regarding allocation and blinding. Also, most of the studies did not report treatment adherence (a factor for poor treatment outcomes) or comorbidities such as HIV/AIDS that can affect treatment outcomes. Other confounders such as age, gender, and previous treatment should be also considered in the analysis. These limitations can affect (in any direction and in magnitude) the measures of associations reported in primary studies. Another limitation of primary studies was the heterogeneous report of treatment outcomes, so a proper comparison between treatments was very difficult, e.g. almost a third part of treatment outcome evaluation was with different clinical scores, which applied diverse items and can be affected by evaluator bias. Future studies evaluating clinical effectiveness of leprosy treatment should be use standard treatment outcomes, with laboratory definitions and/or biomarkers of success, with the aim to compare different settings and clinical characteristics.

Finally, none of these studies reported antibiotic resistance pattern of *M. leprae*. There are reports on drug resistance in leprosy patients [45–49] and can be one of the reasons of the relapse rate increase in the last years [50]. Considering some of the areas with leprosy had also high incidence of resistant tuberculosis (such us India) and MDT share rifampin as an important drug with sensitive tuberculosis, closer follow-up and drug resistance studies should be done in these areas.

However, it is important to mention that studies with people with leprosy are conducted in low resources settings where sophisticated methods of follow-up are not possible, due to lack of money and interest of higher authorities.

### Implications for practice and research

As a stigmatizing disease for centuries, leprosy is nowadays affecting vulnerable populations in the tropics (especially in rural and semi-rural areas), where health systems are weak and other diseases (such as dengue, malaria, HIV/AIDS or tuberculosis) have more preponderance in public health funding [51, 52]. Health determinants affects in several areas (such as poverty, education, water and sanitation, gender and others), reason why treatment alone is not the most effective way to eliminate the disease [53]. In addition, leprosy control faces many barriers regarding clinical management, starting with the lack of funding for research, its characteristic as a silent disease, and unavailable measures for prevention (i.e. an effective vaccine). A delayed diagnosis and treatment usually lead to disability, which regardless the success of treatment, will lead to the necessity of access to health care and rehabilitation for long time, sometimes lifelong [54]. In addition, there are not new drugs in the pipeline to reduce treatment duration, and the only drug in research phase is bedaquiline (which is also one of the new drugs for tuberculosis after more than 30 years of tuberculosis treatment), which showed a bactericidal effect against *M. leprae* in animal models [55, 56]. Now, Bedaquiline is being tested in a Phase 2 trial (not started yet) for MB leprosy in Brazil [57], however, as in the case of tuberculosis, we need new drugs that can be combined to reduce the burden of *M. leprae*, specially in an context of increasing antibiotic resistance, providing a safe and short leprosy treatment.

## Conclusions

None of the evaluated regimes showed any benefit over MDT for patients with PB or MB for relapses. The addition of clofazimine to PB MDT did not show significant improvement.

It is necessary to standardize criteria for diagnosis, cure, and follow-up in the search for more and better evidence to fill existing gaps in information that evaluates the new available antibiotics.

Further studies evaluating adherence to the treatment, potential development of drug resistance and short treatment regimens based in evidence are needed to reach the goal of leprosy elimination.

## Supplementary information

**Additional file 1.** Search strategy. Search strategies for the different databases.

**Additional file 2.** Quality assesment using the Newcastle- Ottawa scale. Quality assessment for non-RCT studies using the Newcastle-Ottawa scale.

## Data Availability

Maria Lazo-Porras and Gabriela J. Prutsky had full access to all the data in the study and take responsibility for the integrity of the data and the accuracy of the data analysis. Data will be provided under request to the first authors.

## Abbreviations

BI: Bacillary Index
CI: Confidence Intervals
ENL: Erythema Nodosum Leprosorum
HIV/AIDS: Human Immunodeficiency Virus/Acquired Immune Deficiency Syndrome
MB: Multibacillary
MD: Mean difference
MDT: Multidrug Therapy
NHDP: National Hansen’s Diseases Programs
PB: Paucibacillary
RCT: Randomized Controlled Trials
ROM: Rifampin-Ofloxacin-Minocycline
RR: Relative Risk
SDM: Standardized Mean Difference
TB: Tuberculosis
WHO: World Health Organization
