# Supplementary material for: World Health Organization (WHO) antibiotic regimen against other regimens for the treatment of leprosy: a systematic review and meta-analysis
Source: BMC Infect Dis. 2020 Jan 20;20:62. doi: 10.1186/s12879-019-4665-0 (PMC6971933; doi:10.1186/s12879-019-4665-0)
Supplement: Supplementary file 1 — Additional file 1. Search strategy. Search strategies for the different databases. [file 12879_2019_4665_MOESM1_ESM.docx]

# Additional file 1. Search strategy

Ovid

Database(s): Embase 1988 to 2011 Week 26, Ovid MEDLINE(R) In-Process & Other Non-Indexed Citations and Ovid MEDLINE(R) 1948 to Present, EBM Reviews - Cochrane Central Register of Controlled Trials 2nd Quarter 2011, EBM Reviews - Cochrane Database of Systematic Reviews 2005 to June 2011
Search Strategy:

| **#** | **Searches** | **Results** |
| --- | --- | --- |
| 1 | exp Leprosy/dt [Drug Therapy] | 7237 |
| 2 | (leprosy or "hansen disease" or "hansen's disease").mp. [mp=ti, ab, sh, hw, tn, ot, dm, mf, dv, kw, ps, rs, nm, ui, tx, ct] | 33026 |
| 3 | exp world health organization/ | 69477 |
| 4 | (world health organization or who).mp. [mp=ti, ab, sh, hw, tn, ot, dm, mf, dv, kw, ps, rs, nm, ui, tx, ct] | 2503316 |
| 5 | (mdt or "multi-drug therapy" or "multidrug therapy").mp. [mp=ti, ab, sh, hw, tn, ot, dm, mf, dv, kw, ps, rs, nm, ui, tx, ct] | 4085 |
| 6 | (3 or 4) and 5 | 1272 |
| 7 | (1 or 2) and 6 | 904 |
| 8 | exp controlled study/ | 3557939 |
| 9 | exp evidence based medicine/ | 496393 |
| 10 | evidence-based.mp. | 166738 |
| 11 | ((control$ or randomized) adj2 (study or studies or trial or trials)).mp. [mp=ti, ab, sh, hw, tn, ot, dm, mf, dv, kw, ps, rs, nm, ui, tx, ct] | 4552383 |
| 12 | meta analysis/ | 83285 |
| 13 | meta-analys$.mp. | 131808 |
| 14 | exp "systematic review"/ | 41661 |
| 15 | systematic review$.mp. | 92077 |
| 16 | exp Guideline/ or exp Practice Guideline/ | 263526 |
| 17 | guideline$.ti. | 83932 |
| 18 | or/8-17 | 5049756 |
| 19 | exp Cohort Studies/ | 1286068 |
| 20 | exp longitudinal study/ | 854233 |
| 21 | exp retrospective study/ | 601324 |
| 22 | exp prospective study/ | 512572 |
| 23 | exp observational study/ | 21043 |
| 24 | exp comparative study/ | 2146789 |
| 25 | exp clinical trial/ | 1443237 |
| 26 | exp evaluation/ | 1056039 |
| 27 | exp validation study/ | 25445 |
| 28 | ((clinical or evaluation or validation or pilot or comparative or cohort or longitudinal or retrospective or prospective or concurrent or follow-up or observational) adj (study or studies or analysis or analyses or trial or trials)).mp. | 6281412 |
| 29 | or/19-28 | 6887560 |
| 30 | 7 and (18 or 29) | 508 |
| 31 | from 7 keep 436-862 | 427 |
| 32 | limit 31 to (clinical trial, all or clinical trial, phase i or clinical trial, phase ii or clinical trial, phase iii or clinical trial, phase iv or clinical trial or comparative study or controlled clinical trial or evaluation studies or guideline or meta analysis or multicenter study or practice guideline or randomized controlled trial or validation studies) [Limit not valid in Embase,CCTR,CDSR; records were retained] | 87 |
| 33 | 30 or 32 | 508 |
| 34 | limit 33 to (book or book series or editorial or erratum or letter or addresses or autobiography or bibliography or biography or comment or dictionary or directory or interactive tutorial or interview or lectures or legislation or news or newspaper article or patient education handout or periodical index or portraits or published erratum or video-audio media or webcasts) [Limit not valid in Embase,Ovid MEDLINE(R),Ovid MEDLINE(R) In-Process,CCTR,CDSR; records were retained] | 20 |
| 35 | 33 not 34 | 488 |
| 36 | limit 35 to human [Limit not valid in CCTR,CDSR; records were retained] | 475 |
| 37 | limit 36 to humans [Limit not valid in CCTR,CDSR; records were retained] | 475 |
| 38 | from 7 keep 863-904 | 42 |
| 39 | 37 or 38 | 494 |
| 40 | limit 39 to yr="1982 -Current" | 494 |
| 41 | remove duplicates from 40 | 318 |

Scopus

1. TITLE-ABS-KEY(leprosy or "hansens disease" or "hansen's disease") AND PUBYEAR AFT 1981
2. TITLE-ABS-KEY("world health organization" or who)
3. TITLE-ABS-KEY(mdt or "multi-drug therapy" or "multidrug therapy")
4. 1 and 2 and 3
5. TITLE-ABS-KEY("comparative study" OR "comparative survey" OR "comparative analysis" OR "cohort study" OR "cohort survey" OR "cohort analysis" OR "longitudinal study" OR "longitudinal survey" OR "longitudinal analysis" OR "retrospective study" OR "retrospective survey" or "retrospective analysis" OR "prospective study" OR "prospective survey" OR "prospective analysis" OR "concurrent study" OR "concurrent survey" OR "concurrent analysis" or "follow-up study" OR "follow-up survey" OR "follow-up analysis" or "observational study" OR "observational survey" OR "observational analysis" OR "clinical study" OR "clinical trial" or "evaluation study" OR "evaluation survey" OR "evaluation analysis" or "validation study" OR "validation survey" OR "validation analysis")
6. TITLE-ABS-KEY( (evidence W/1 based) OR (meta W/1 analys*) OR (systematic* W/2 review*) OR guideline OR (control* W/2 stud*) OR (control* W/2 trial*) OR (randomized W/2 stud*) OR (randomized W/2 trial*))
7. 4 and (5 or 6)
8. PMID(0*) OR PMID(1*) OR PMID(2*) OR PMID(3*) OR PMID(4*) OR PMID(5*) OR PMID(6*) OR PMID(7*) OR PMID(8*) OR PMID(9*)
9. 7 and not 8
10. DOCTYPE(le) OR DOCTYPE(ed) OR DOCTYPE(bk) OR DOCTYPE(er) OR DOCTYPE(no) OR DOCTYPE(sh)
11. 9 and not 10

LILACS

Keywords Searched

leprosy or hansens disease or hansen's disease [Words]

and who or world health organization [Words]

and therapy or mdt [Words]
