# Supplementary material for: World Health Organization (WHO) antibiotic regimen against other regimens for the treatment of leprosy: a systematic review and meta-analysis
Source: BMC Infect Dis. 2020 Jan 20;20:62. doi: 10.1186/s12879-019-4665-0 (PMC6971933; doi:10.1186/s12879-019-4665-0)
Supplement: Supplementary file 2 — Additional file 2. Quality assesment using the Newcastle- Ottawa scale. Quality assessment for non-RCT studies using the Newcastle-Ottawa scale. [file 12879_2019_4665_MOESM2_ESM.docx]

**Additional file 2. Quality assesment using the Newcastle- Ottawa scale**

| **Author, year** | **Type of study** | **Selection** | | | | **Comparability** | **Outcome** | | |
| --- | --- | --- | --- | --- | --- | --- | --- | --- | --- |
|  |  | **Representativeness of the exposed cohort** | **Selection of the non-exposed cohort** | **Ascertainment of exposure** | **Demonstration that outcome of interest was not present at start of study** | **Comparability of cohorts on the basis of the design or analysis** | **Assessment of outcome** | **Was follow-up long enough for outcomes to occur** | **Adequacy of follow up of cohorts** |
| Balagón, 2011 | Cohort | selected group of users (Volunteers) | Drawn from a different source (same place different years) | secure record | yes | Comparability of cohorts on the basis of the design or analysis study controls for treatment | Record linkage | no | No statement |
